# The influence of children’s theory of mind and sibling relationship quality on sibling teaching in the Chinese family with two children

**DOI:** 10.3389/fpsyg.2026.1751491

**Published:** 2026-02-11

**Authors:** Xiaojun Cao, Linhan Pang

**Affiliations:** 1 College of Psychology, Sichuan Normal University, Chengdu, China; 2 School of Education, China West Normal University, Nanchong, China

**Keywords:** educational psychology, family education, sibling relationship quality, sibling teaching, theory of mind

## Abstract

**Introduction:**

Sibling teaching provides an important context for children’s cognitive and social development, particularly in families with more than one child. Within the Chinese family context shaped by the two-child policy, older siblings often assume instructional roles. However, limited research has examined how children’s theory of mind and sibling relationship quality jointly influence instructional behaviors and learning engagement during sibling teaching interactions. This study aimed to address this gap by examining these associations across different instructional task types.

**Methods:**

Thirty-four sibling dyads from Chinese two-child families participated in structured teaching tasks, including mathematical/spatial and language activities. Firstborn children (aged 6–12 years) acted as instructors, while second-born children (aged 3–6 years) acted as learners. Children’s theory of mind was assessed using standardized tasks, and sibling relationship quality was reported by parents. Teaching and learning behaviors were coded from video-recorded interactions using an established observational coding system. Multiple regression analyses were conducted to examine predictive relationships.

**Results:**

Results showed that instructors’ theory of mind positively predicted the use of cognitive strategies and positive feedback across both mathematical/spatial and language teaching tasks. Learners’ Theory of Mind was negatively associated with off-task behavior in both task types and with refusal behavior in language teaching tasks. Sibling relationship quality positively predicted instructors’ cognitive strategies and learners’ verbal and physical engagement, with stronger effects observed in mathematical/spatial teaching tasks.

**Discussion:**

These findings highlight the joint roles of social–cognitive development and sibling relationship quality in shaping sibling teaching interactions. By distinguishing between task types and examining both instructor and learner behaviors, the study extends existing theories of sibling teaching and underscores the importance of family-based learning processes within a Chinese cultural context.

## 1 Introduction

### 1.1 Research background

Children living in a family environment with at least two children have more opportunities to promote each other’s cognitive development (Dunn, 2002; Howe and Recchia, 2009). Teaching is a conscious and purposeful activity (Rogoff, 1998; Segal et al., 2018). Although unconscious teaching behaviors also frequently occur in sibling interactions, this article discusses conscious and purposeful sibling teaching. Thus, based on the definition of teaching and prior literature, purposeful and conscious sibling teaching occurs when one child decides to systematically impart their knowledge to a sibling, and the sibling actively participates in the teaching process. In this phenomenon, the sibling with more knowledge and skills (the teacher) intentionally transfers knowledge and skills to the sibling with less knowledge and skills (the learner) through guidance and direct instruction. This is known as sibling teaching.

With the comprehensive promotion of China’s two-child policy, more and more families are having a second child, leading to an increase in multi-child families, which is becoming an increasingly common family structure in China (Zhao and Yu, 2017; Jiang et al., 2022). How siblings interact with each other has therefore become a growing concern for parents. Negative events such as sibling conflicts can be troubling for parents and may pose challenges for family functioning (Brody, 1998). However, if parents can appropriately guide their children and facilitate teaching activities between them, creating a positive environment that encourages frequent teaching interactions, this may improve the quality of sibling relationships and enhance children’s cognitive development (Howe and Recchia, 2009). Analyzing the characteristics and influencing factors of sibling teaching behavior can thus help reveal the positive role of sibling interaction in children’s learning and socialization (Dunn, 2002; Howe and Recchia, 2009). This, in turn, can encourage parents and educators to focus on family education and strive to create supportive environments for positive sibling interactions. However, such implications should be interpreted with caution, as the present study focuses on structured teaching interactions within a specific age range. Accordingly, the present study aims to examine how children’s theory of mind and sibling relationship quality jointly influence instructional behaviors and learning engagement during sibling teaching interactions in Chinese two-child families.

Theory of mind is the ability to understand and explain human behavior and social situations, and to be aware of and comprehend the potential mental states of others, such as knowledge, thoughts, beliefs, emotions, desires, and intentions (Wellman and Liu, 2004; Ziv et al., 2016). It is a core factor in the development of children’s social cognition (Ziv et al., 2016). Once children acquire theory of mind abilities, they can communicate and interact more effectively with others, and the opportunities to interact with siblings and peers and resolve conflicts increase (Gopnik and Astington, 1988; McAlister and Peterson, 2006). Within a certain age range, interactions between siblings can produce unique social experiences that help accelerate the development of children’s theory of mind (McAlister and Peterson, 2006).

Theory of Mind plays a critical role in children’s understanding of others’ mental states and social behaviors (Happé et al., 1998; Liu and Li, 2010; Liu et al., 2008; Du, 2020), and theory of mind is related to children’s teaching strategies (Ziv and Frye, 2004). Similarly, the learner’s theory of mind also impacts their behavior during the teaching process. The learner’s theory of mind facilitates the teacher’s understanding of the dynamics of successful teaching, as it requires the teacher to flexibly impart knowledge and appropriately adjust their teaching strategies (Howe et al., 2019). Whether the influence of theory of mind on teachers and learners is applicable in the Chinese context is an area this paper aims to explore.

The term “sibling relationship” refers to all interactions between brothers and sisters in which they share knowledge, perspectives, attitudes, beliefs, and feelings related to each other. These interactions are characterized by equality, reciprocity, and complementarity (Zhao and Yu, 2017). It is a long-lasting, relatively stable, and non-voluntary interpersonal relationship throughout one’s life. Additionally, in families with more than one child, sibling relationships are an important branch of the family network. According to Zhao and Yu (2017), the academic field has not yet reached a unified definition of the concept of sibling relationship quality, nor is there a theory that comprehensively explains it. However, sibling relationship quality can generally be divided into two dimensions: sibling warmth and sibling conflict. From a psychological perspective, sibling relationship quality can be categorized into two aspects: positive sibling relationships and negative sibling relationships.

Previous research has confirmed the impact of sibling relationship quality on the behavior of both educators and learners. According to Howe and Recchia (2005), when educators perceive sibling relationships positively, they are more likely to encourage learners to actively engage in teaching activities and use fewer control strategies in their teaching. Learners who hold a positive view of sibling relationships are more likely to employ imitative learning methods in their educational activities (Howe and Recchia, 2009). The applicability of these conclusions in the Chinese context is also worth exploring. This study aims to investigate the impact of sibling relationship quality on educators and learners within the Chinese environment.

Beyond examining the applicability of prior findings in a Chinese context, the present study seeks to refine theoretical understanding of sibling teaching by distinguishing between different types of teaching behaviors and by examining whether the roles of theory of mind and sibling relationship quality vary across instructional tasks. Importantly, the Chinese family context, characterized by stronger hierarchical sibling roles and greater emphasis on intra-family teaching, provides a theoretically meaningful context to examine whether and how established social–cognitive mechanisms of sibling teaching operate under different cultural expectations. The present study makes several contributions to the literature on sibling teaching and social–cognitive development. First, extending prior research that has predominantly focused on mathematical or spatial problem-solving tasks, this study incorporates a language teaching task, thereby broadening the scope of sibling teaching research to include verbal instructional contexts. Second, the language teaching task is based on Chinese character learning, which differs fundamentally from alphabetic systems and involves distinct cognitive and instructional demands. This allows for a more culturally grounded examination of teaching and learning processes within Chinese families. Third, by comparing mathematical/spatial and language teaching tasks, this study demonstrates that the roles of children’s theory of mind and sibling relationship quality vary across instructional contexts, highlighting task type as an important boundary condition in sibling teaching processes. Together, these contributions move beyond simple cultural replication and provide a more differentiated understanding of how social–cognitive and relational factors jointly shape sibling teaching behaviors in Chinese two-child families.

### 1.2 Research hypotheses

*H1*: In sibling teaching contexts, the higher the level of instructor’s theory of mind (firstborn child), the more positively it predicts the frequency of their use of cognitive strategies.

*H2*: In sibling teaching contexts, the higher the level of learner’s theory of mind (second born child), the more positively it predicts the frequency of their use of verbal expression and physical expression and the lower their off-task behavior.

*H3*: Higher sibling relationship quality positively predicts the use of cognitive strategies and positive feedback by the educator, as well as the frequency of verbal expression and physical expression by the learner.

*H4*: Lower sibling relationship quality positively predicts the use of negative feedback by the educator.

## 2 Method

### 2.1 Subjects

A random sample of 34 families with two children was selected for the present study. Families were recruited through kindergartens and primary schools in several cities in China, using convenience sampling. Eligible families who expressed interest were included in the recruitment pool, and sibling dyads meeting the age criteria were then randomly selected from this pool to participate in the study. Among these families, 6 pairs of siblings were from Chengdu, Sichuan Province; 5 pairs from Nanchong, Sichuan Province; 5 pairs from Chongqing; and the remaining 18 pairs from Dazhou, Sichuan Province. A total of 68 children participated in the study (31 boys and 37 girls). In this study, firstborn children (*M* = 8.68 years, SD = 1.90) assumed the role of the educator, whereas second-born children (*M* = 4.59 years, SD = 1.08) assumed the role of the learner. According to the combinations of gender and birth order, the sample included 17 same-gender sibling pairs (7 brother–brother pairs and 10 sister–sister pairs) and 17 opposite-gender sibling pairs (8 brother–sister pairs and 9 sister–brother pairs). The age gap between siblings ranged from 0 to 9 years (*M* = 4.09 years, SD = 2.30), with a 0-year gap indicating twin pairs.

The age ranges were selected to reflect typical instructor–learner roles in sibling teaching contexts. Firstborn children aged 6–12 years generally possess more advanced cognitive and communicative abilities, enabling them to assume instructional roles, whereas second-born children aged 3–6 years are at a developmental stage where guided learning from older siblings is common. This age configuration is consistent with prior sibling teaching research.

### 2.2 Research procedure

During the experimental study, each family served as a unit, and the experiment was conducted a total of 34 times (visiting 34 families). In each family’s experimental study, the experimental content of Study 2 was conducted first, followed by the experimental content of Study 1. The general procedure is as follows: First, the “Sibling Relationship Questionnaire for Chinese Preschool Children (Parental Version)” was provided to the parents of the two children, who were asked to fill out the questionnaire. Meanwhile, both children underwent separate cognitive theory tests. After completing the cognitive theory tests, and following a brief rest, the firstborn child was taken aside to familiarize themselves with the teaching materials and process. Once the firstborn child was prepared, the firstborn child and the second-born child engaged in a sibling teaching activity, starting with a verbal teaching activity followed by a math/spatial teaching activity. The entire process of the sibling teaching activity was recorded. The researcher then coded the behaviors of the educator and learner for subsequent data statistical analysis.

### 2.3 Research tools

#### 2.3.1 Sibling teaching

The materials used for sibling teaching activities were divided into math/spatial teaching materials and verbal teaching materials. Based on experiences from foreign research methodologies and the “Guidelines for Children’s Learning and Development (Ages 3–6),” LEGO was selected as the teaching material for math/spatial knowledge in this study. The researcher conducted surveys in several kindergartens, drawing on the experiences of frontline kindergarten teachers. Larger LEGO pieces were chosen as they are suitable for 3–6-year-old children to grasp and assemble. The goal tasks for assembling LEGO were set as simple and complex teaching tasks. Under the guidance of the firstborn child, the second-born child assembled LEGO according to the instructions provided in the given images. Details of the target images for simple and complex teaching tasks can be found in Appendix A.

The verbal teaching activities in this study mainly utilized the “Game-based Literacy Method.” The design of the teaching materials was based on the “Guidelines for Children’s Learning and Development (Ages 3–6)” and incorporated the results of the researcher’s surveys in several kindergartens, drawing on the experiences of frontline kindergarten teachers. This was combined with some children’s word game books to design simple and complex tasks for verbal knowledge suitable for the children’s zone of proximal development. Under the guidance of the firstborn child, the second-born child matched the pictures with the corresponding Chinese character cards. The goal of the simple teaching task was to match the Chinese character cards with the corresponding pictures. The goal of the complex teaching task was to match the Chinese character cards, the idiom and the ancient poems with the corresponding pictures. Details of the simple and complex teaching tasks are provided in Appendix B.

The mathematical/spatial and language tasks were designed to be age-appropriate and flexible in difficulty, allowing instructors to adjust their teaching strategies according to the learner’s level. The use of two task domains also enabled examination of whether instructional behaviors varied across different learning contexts. The behaviors of the instructor and learner during the sibling teaching process were recorded using a video camera, and these behaviors were later classified and coded. Based on the coding and specific definitions of the behaviors of instructors and learners by Howe et al. (2006), Howe and Recchia (2009), and Howe et al. (2019), some localized modifications were made according to the actual situation, as detailed below:

1. Instructor behavior coding standards.

**Table:** 

Physical demonstrations	The instructor demonstrates the operation of teaching aids, such as sequentially showcasing them to the learners or personally demonstrating how to place or assemble a LEGO piece. The learners observe the demonstration without actively participating.
Cognitive strategies	The instructor adopts a learner-centered approach, allowing learners to engage in hands-on activities while providing scaffolded instruction. The instructor follows a scaffolded teaching model that involves the following: Providing prompts or clues to guide learners: The instructor offers hints, prompts, or cues to help learners navigate the task or problem. Motivating, guiding, or instructing learners to work independently: The instructor encourages learners to take ownership of their learning, providing guidance and support as needed while fostering independent problem-solving skills. Offering explanations to learners: The instructor provides explanations, clarifications, or demonstrations to enhance learners’ understanding of concepts, procedures, or strategies. Providing instructions for learners to continue with the task: The instructor offers instructions or prompts for learners to proceed with the task, ensuring a smooth progression and facilitating their ongoing engagement and learning.
Descriptions	The instructor narrates their own actions during the teaching process or solely provides instructions on how to complete the task, while the learners observe without actively participating. The instructor reads the text from the instructional materials without considering whether the learners have understood or mastered the content. The learners passively observe while the instructor reads.
Positive feedback	The instructor provides positive feedback, encouragement, or praise in an appreciative or neutral tone. For example, the instructor might say, “Yes, that’s correct!” or “You did a great job!” The instructor utilizes specific physical actions to express praise and encouragement, such as clapping hands or giving a thumbs-up gesture.
Control/Negative feedback	The instructor identifies and corrects learners’ errors in a dictatorial or impatient tone or forcibly compels learners to engage in the instructional activity. For example, the instructor may shout at the learners, “You’re so stupid!” or “I told you to hurry up!” The instructor resorts to aggressive physical actions to correct or restrain learners, even resorting to physically attacking them.
Off task	The instructor becomes distracted by external irrelevant stimuli, such as engaging in conversations with researchers or getting involved in activities unrelated to the teaching task. The instructor solely focuses on completing their own tasks without engaging in communication with the learners or paying attention to them, completely disregarding the original intention of “teaching.”

1. Learner behavior coding standards

**Table:** 

Verbal expression	Learners engage in verbal imitation. Learners respond to the instructor’s questions or narrate learning content as per the instructor’s instructions. Learners seek guidance from the instructor when facing difficulties.
Physical expression	Learners manipulate instructional materials under the guidance of the instructor or independently.
Positive feedback	Learners actively respond to the instructor, express appreciation, or comply with instructions. For example, learners may say phrases like “That’s right” or “Okay!” to show their agreement or willingness to follow the instructions.
Control/Negative feedback	Learners display an authoritarian or impatient tone when interacting with the instructor. Learners become angry or physically aggressive towards the instructor. Learners engage in conflicts with the instructor over the possession or control of instructional materials during the teaching process.
Refusal	Learners refuse the instructor’s attempt to impart knowledge to them and express their refusal verbally, for example, telling the instructor, “No, I do not need you to teach me, I can do it myself!” Learners physically demonstrate their refusal, such as pushing the instructor’s hand aside.
Off -task	Learners become distracted by external irrelevant stimuli, such as engaging in conversations with researchers or completely disregarding the instructor’s instructions and focusing on manipulating instructional materials on their own.

Ethical review and approval was not required for this study in accordance with local legislation and institutional requirements. The study involved non-invasive, minimal-risk observational procedures conducted in familiar and naturalistic settings. Parents or legal guardians were fully informed about the purpose and procedures of the study prior to participation and provided verbal consent for their children’s participation and video recording. Video recordings were used solely for behavioral coding and analysis, were anonymized during data processing, and were not intended for public sharing.

#### 2.3.2 Theory of mind

The specific questions for testing the theory of mind of the two children are detailed in Appendix C. First, the test tool for assessing the theory of mind of the firstborn child consists of two parts, with a total of 5 tasks:

1. Belief-desire reasoning task:

The experimental materials were adapted from the research paradigm of Leslie et al. (2005), with further adaptations by Liu and An (2010) and Du (2020). There are a total of 4 stories. In this task, correctly answering the control question, desire question, second-level belief-desire reasoning question, and confirmation question earns 1 point; otherwise, it earns 0 points. Thus, the score range for each participant is 0 to 1 point.

1. Understanding Blunders Task:

Based on the blunder recognition task paradigm designed by Stone et al. (1998), it was localized and adapted by Liu and An (2010). The experimental material consists of a single blunder story. After reading the story, the participant is asked two questions. The first question mainly examines whether the participant can correctly judge whether someone in the story made a blunder. If the participant answers incorrectly, the second question is not asked, and they are given a score of 0. If the participant answers correctly, the second question is asked: “Who was the person that made the blunder?” If the participant can correctly identify which character made the blunder (i.e., answers the second question correctly), they are awarded 1 point. Answering all questions correctly results in a total score of 2 points.

Secondly, the test tool for assessing the theory of mind of the secondborn child consists of three tasks, with a final score ranging from 0 to 6 points.

1. Unexpected Location Task:

This task uses the Sally-Anne test developed by Baron-Cohen et al. (1985), which was localized and modified by Ge et al. (2021). The detection question is not scored, while the false belief question and behavior prediction question are each scored on a 0/1 basis. The final score for this task is between 0 and 2 points.

1. Unexpected Content Task:

This task uses content adapted by Ge et al. (2021) from the research paradigm of Gopnik and Astington (1988). The researcher presents the test content to the participating children through physical demonstrations. The detection question is not scored, while the representational change question and the false belief question are each scored on a 0/1 basis. The final score for this task is between 0 and 2 points.

1. Appearance-Reality Distinction Task:

This task uses content adapted by Ge et al. (2021) from the research paradigm of Gopnik and Astington (1988). The researcher presents the test content through physical demonstrations. The detection question is not scored, while the representational change question and the false belief question are each scored on a 0/1 basis. The final score for this task is between 0 and 2 points.

#### 2.3.3 Sibling relationship quality

The Sibling Relationship Questionnaire for Chinese Preschool Children (Parental Version) was developed by Jiang et al. (2022). This questionnaire consists of four dimensions: Sibling Interaction, Sibling Acceptance, Sibling Intimacy, and Sibling Rivalry, with a total of 18 items: 5 items for Sibling Interaction, 4 items for Sibling Acceptance, 5 items for Sibling Rivalry, and 4 items for Sibling Intimacy. The overall questionnaire and the internal consistency coefficients for each dimension range from 0.759 to 0.8548, and the *α* coefficients for each dimension range from 0.759 to 0.810.

Specifically, the Sibling Intimacy dimension measures the level of warmth between siblings, for example, “Dabao will protect his/her younger brother/sister when they are afraid.” The Sibling Acceptance dimension assesses the degree of acceptance Dabao shows toward his/her younger brother/sister, for example, “Dabao will show off his brother/sister to others.” The Sibling Rivalry dimension primarily reflects the level of conflicts between siblings, for example, “Dabao gets angry when his brother/sister breaks his things.” The Sibling Interaction dimension focuses on the interaction between siblings, for example, “Dabao and his brother/sister tell each other their little secrets.” Each item in the questionnaire is scored on a 5-point scale: 1 = Never happens, 2 = Rarely happens, 3 = Sometimes happens, 4 = Frequently happens, 5 = Always happens. Items 3, 7, 10, 13, and 17 are reverse scored. Please refer to Appendix C for the specific content of the questionnaire. The specific content of the questionnaire is detailed in Appendix D.

#### 2.3.4 Data management and statistical analysis

Statistical software SPSS 25.0 was used for data management and statistical analysis.

## 3 Results

### 3.1 Internal consistency testing

#### 3.1.1 The instructional behaviors of the instructor

The instructor’s behaviors encompass six categories. However, based on the actual conditions of the experiment, the instructors rarely engage in off-task behaviors related to teaching. Therefore, the encoded instructor behaviors for analysis consist of five categories: physical demonstration, cognitive strategies, description, positive feedback, and negative feedback. Two coders coded the instructor’s behaviors and calculated their frequencies. Subsequently, the internal consistency of the coding was computed, resulting in:

First, in simple mathematics/spatial teaching tasks, the Cohen’s kappa coefficient for physical demonstration is 0.91, for description is 0.90, for cognitive strategies is 0.88, for positive feedback is 1.00, and for negative feedback is 1.00. Second, in complex mathematics/spatial teaching tasks, the Cohen’s kappa coefficient for physical demonstration is 0.91, for description is 0.93, for cognitive strategies is 0.88, for positive feedback is 1.00, and for negative feedback is 1.00. Third, in simple language teaching tasks, the Cohen’s kappa coefficient for physical demonstration is 0.97, for description is 0.96, for cognitive strategies is 0.91, for positive feedback is 1.00, and for negative feedback is 1.00. Fourth, in complex language teaching tasks, the Cohen’s kappa coefficient for physical demonstration is 0.97, for description is 0.93, for cognitive strategies is 0.88, for positive feedback is 1.00, and for negative feedback is 1.00.

#### 3.1.2 The behaviors of the learners

The behaviors of the learners can be categorized into six types: verbal expression, physical expression, refusal, positive feedback, negative feedback, and off-task behavior. Two coders were responsible for coding the learners’ behaviors, and their frequencies were calculated. Subsequently, the internal consistency of the coding was assessed, resulting in the following outcomes:

First, in simple mathematics/spatial teaching tasks, the Cohen’s kappa coefficient for verbal expression is 0.94, for physical expression is 0.90, for refusal is 1.00, for positive feedback is 1.00, for negative feedback is 1.00, and for off-task behavior is 0.95. Second, in complex mathematics/spatial teaching tasks, the Cohen’s kappa coefficient for verbal expression is 0.94, for physical expression is 0.85, for refusal is 1.00, for positive feedback is 1.00, for negative feedback is 1.00, and for off-task behavior is 0.95. Third, in simple language teaching tasks, the Cohen’s kappa coefficient for verbal expression is 0.97, for physical expression is 0.97, for refusal is 1.00, for positive feedback is 1.00, for negative feedback is 1.00, and for off-task behavior is 1.00. Fourth, in complex language teaching tasks, the Cohen’s kappa coefficient for verbal expression is 0.91, for physical expression is 0.97, for refusal is 1.00, for positive feedback is 1.00, for negative feedback is 1.00, and for off-task behavior is 0.95.

### 3.2 The impact of theory of mind on instructor’s behavior

#### 3.2.1 The relationship between instructor’s theory of mind and behavior

In the math/spatial teaching task and the language teaching task, Pearson correlation analyses were conducted to examine the relationship between the theory of mind (TOM) scores of the instructors and their five types of behavior. The results are presented in Table 1. It can be summarized that in the math/spatial teaching task, the theory of mind scores of the instructors were significantly positively correlated with their cognitive strategies (*r* = 0.70, *p* < 0.001) and positive feedback (*r* = 0.55, *p* < 0.01). In the language teaching task, the theory of mind scores of the instructors were significantly positively correlated with their cognitive strategies (*r* = 0.66, *p* < 0.001) and positive feedback (*r* = 0.41, *p* < 0.05).

**Table 1:** Correlation between instructor’s theory of mind and behavior.

Task type	Predictor	Physical demonstration	Description	Cognitive strategy	Positive feedback	Negative feedback
Math	TOM	−0.21	0.02	0.70***	0.55**	−0.29
Language	TOM	0.07	−0.12	0.66***	0.41*	−0.22

* indicates *p* < 0.05, ** indicates *p* < 0.01, and *** indicates *p* < 0.001.

#### 3.2.2 Predictive role of theory of mind on instructor’s behavior

In the math/spatial teaching task and the language teaching task, the behaviors of the instructor that were significantly correlated with their theory of mind test scores were entered into regression equations. In the math/spatial teaching task, Theory of Mind of the instructor was entered as the predictor variable, and their cognitive strategies and positive feedback were the dependent variables. In the language teaching task, Theory of Mind of the instructor was entered as the predictor variable, and their cognitive strategies and positive feedback were the dependent variables. The results obtained from multiple linear regression analyses are presented in Table 2. The following conclusions can be drawn:

**Table 2:** Predictive role of theory of mind on instructor’s behavior.

Task type	Predictor variable	Dependent variable	*β*	*T*	*△R^2^*	*F*
Math tasks	TOM	Cognitive strategy	0.70	5.46***	0.48	29.84***
	TOM	Positive feedback	0.55	3.73**	0.30	13.89**
Language tasks	TOM	Cognitive strategy	0.66	4.96***	0.44	24.61***
	TOM	Positive feedback	0.41	2.53*	0.17	6.41*

* indicates *p* < 0.05, ** indicates *p* < 0.01, and *** indicates *p* < 0.001.

First, in the math/spatial teaching task, Theory of Mind of the instructor accounted for 48% of the variance in cognitive strategies (*F* = 29.84, *p* < 0.001). Theory of Mind significantly and positively predicted cognitive strategies (*β* = 0.70, *T* = 5.46, *p* < 0.001), indicating that higher levels of Theory of Mind were associated with the use of more cognitive strategies. Furthermore, Theory of Mind of the instructor accounted for 30% of the variance in positive feedback (*F* = 13.89, *p* < 0.01). Theory of Mind significantly and positively predicted positive feedback (*β* = 0.55, *T* = 3.73, *p* < 0.01), suggesting that higher levels of Theory of Mind were associated with the use of more positive feedback.

Second, in the language teaching task, Theory of Mind of the instructor accounted for 44% of the variance in cognitive strategies (*F* = 24.61, *p* < 0.001). Theory of Mind significantly and positively predicted cognitive strategies (*β* = 0.66, *T* = 4.96, *p* < 0.001), indicating that higher levels of Theory of Mind were associated with the use of more cognitive strategies. Additionally, Theory of Mind of the instructor accounted for 17% of the variance in positive feedback (*F* = 6.41, *p* < 0.05). Theory of Mind significantly and positively predicted positive feedback (*β* = 0.41, *T* = 2.53, *p* < 0.05), suggesting that higher levels of Theory of Mind were associated with the use of more positive feedback. Taken together, these results indicate that instructors’ theory of mind was positively associated with the use of cognitive strategies and positive feedback during sibling teaching, with consistent effects observed across both math/spatial and language teaching tasks.

### 3.3 The influence of theory of mind on learner’s behavior

#### 3.3.1 The relationship between learner’s theory of mind and their behavior

In the mathematical/spatial teaching tasks and language teaching tasks, Pearson correlation analyses were conducted to examine the relationship between learners’ scores on the theory of mind test and their 6 behaviors. The results are presented in Table 3. It can be concluded that in the mathematical/spatial teaching tasks, there was a significant negative correlation between learners’ theory of mind test scores and their off-task behavior (*r* = −0.67, *p* < 0.001). In the language teaching tasks, learners’ theory of mind test scores were negatively correlated with their refusal behavior (*r* = −0.44, *p* < 0.05) and off-task behavior (*r* = −0.43, *p* < 0.05).

**Table 3:** Correlation between learner’s theory of mind and behavior.

Task type	Predictor	Verbal expression	Physical expression	Refusal	Positive feedback	Negative feedback	Off-task
Math	TOM	−0.14	−0.07	0.00	−0.21	0.05	−0.67***
Language	TOM	−0.23	−0.17	−0.34	−0.31	−0.12	−0.49**

* indicates *p* < 0.05, ** indicates *p* < 0.01, and *** indicates *p* < 0.001.

#### 3.3.2 The predictive role of learner’s theory of mind on their behavior

In the mathematical/spatial teaching tasks and language teaching tasks, the learner behaviors that were found to have a correlation with the learner’s theory of mind test scores were entered into regression equations. In the mathematical/spatial teaching tasks, the learner’s theory of mind was used as the predictor variable, and off-task behavior was used as the dependent variable. In the language teaching tasks, the learner’s theory of mind was used as the predictor variable, and refusal behavior and off-task behavior were used as the dependent variables. Multiple linear regression analyses were conducted, and the results are presented in Table 4. It can be summarized as follows:

**Table 4:** Predictive effects of learner’s theory of mind on their behavior.

Task type	Predictor variable	Dependent variable	*β*	*T*	*△R^2^*	*F*
Math	TOM	Off-task	−0.67	−5.06***	0.44	25.55***
Language	TOM	Off-task	−0.49	−3.14**	0.24	9.86**

* indicates *p* < 0.05, ** indicates *p* < 0.01, and *** indicates *p* < 0.001.

First, in the mathematical/spatial teaching tasks, the learner’s theory of mind accounted for 44% of the variance in off-task behavior, *F* = 25.55, *p* < 0.001. The theory of mind had a significant negative predictive effect on off-task behavior (*β* = −0.67, *T* = −5.06, *p* < 0.001). Therefore, learners with lower levels of theory of mind exhibited more off-task behavior.

Second, in the language teaching tasks, the learner’s theory of mind accounted for 24% of the variance in off-task behavior, *F* = 9.86, *p* < 0.01. The theory of mind had a negative predictive effect on off-task behavior (*β* = −0.49, *T* = −3.14, *p* < 0.01). Therefore, learners with lower levels of theory of mind exhibited more off-task behavior. Taken together, these results indicate that learners’ theory of mind was consistently and negatively associated with off-task behavior across both mathematical/spatial and language teaching tasks.

### 3.4 The influence of sibling relationship quality on instructor’s behavior

#### 3.4.1 The influence of sibling relationship quality on instructor’s behavior

In the mathematical/spatial teaching tasks and language teaching tasks, the scores obtained from the Sibling Relationship Questionnaire were correlated with the instructor’s five behaviors using Pearson correlation analysis. The results are shown in Table 5. It can be summarized as follows:

**Table 5:** Correlation between sibling relationship quality and instructor’s behavior.

Task type	Predictor	Physical demonstration	Description	Cognitive strategy	Positive feedback	Negative feedback
Math	SRQ	−0.13	−0.12	0.51**	0.23	−0.37*
Language	SRQ	−0.18	−0.23	0.37*	0.05	−0.30

In the mathematical/spatial teaching tasks, sibling relationship quality (SRQ) was significantly positively correlated with the instructor’s cognitive strategies (*r* = 0.51, *p* < 0.01) and negatively correlated with the instructor’s negative feedback (*r* = −0.37, *p* < 0.05). In the language teaching tasks, sibling relationship quality was positively correlated with the instructor’s cognitive strategies (*r* = 0.37, *p* < 0.05).

#### 3.4.2 Predictive role of sibling relationship quality on instructor’s behavior

In the mathematical/spatial teaching task and the language teaching task, instructor behaviors that are correlated with sibling relationship quality were included in the regression equation. In the mathematical/spatial teaching task, sibling relationship quality was used as the predictor variable, and the instructor’s cognitive strategies and negative feedback were the dependent variables. In the language teaching task, sibling relationship quality was used as the predictor variable, and the instructor’s cognitive strategies were the dependent variable. The results obtained from multiple linear regression analysis are presented in Table 6, and the following conclusions can be drawn:

**Table 6:** Predictive effects of sibling relationship quality on instructor’s behavior.

Task type	Predictor variable	Dependent variable	*β*	*T*	*△R^2^*	*F*
Math	SRQ	Cognitive strategy	0.51	3.38**	0.26	11.39**
	SRQ	Positive feedback	−0.37	−2.22*	0.13	4.93*
Language	SRQ	Cognitive strategy	0.37	2.25*	0.14	5.05*

First, in the mathematical/spatial teaching task, the predictor variable of sibling relationship quality accounted for 26% of the variance in cognitive strategies (*F* = 11.39, *p* < 0.01). Sibling relationship quality had a significant positive predictive effect on cognitive strategies (*β* = 0.51, *T* = 3.38, *p* < 0.01), indicating that higher levels of sibling relationship quality were associated with the use of more cognitive strategies by the instructor. Furthermore, the predictor variable of sibling relationship quality accounted for 13% of the variance in negative feedback given by the instructor (*F* = 4.93, *p* < 0.05). Sibling relationship quality had a significant negative predictive effect on negative feedback (*β* = −0.37, *T* = –2.22, *p* < 0.05), indicating that higher levels of sibling relationship quality were associated with less negative feedback from the instructor.

Second, in the language teaching task, the predictor variable of sibling relationship quality accounted for 14% of the variance in cognitive strategies (*F* = 5.05, *p* < 0.05). Sibling relationship quality had a significant positive predictive effect on cognitive strategies (*β* = 0.37, *T* = 2.25, *p* < 0.05), indicating that higher levels of sibling relationship quality were associated with the use of more cognitive strategies by the instructor. Overall, these findings indicate that sibling relationship quality was positively associated with instructors’ use of cognitive strategies and negatively associated with their use of negative feedback, with consistent effects observed across both mathematical/spatial and language teaching tasks.

### 3.5 The impact of sibling relationship quality on learner’s behavior

#### 3.5.1 The relationship between sibling relationship quality and learner’s behavior

In the math/spatial teaching task and the language teaching task, the scores of the Sibling Relationship Quality questionnaire were correlated with the learner’s behaviors using Pearson correlation analysis. The results are shown in Table 7. It can be summarized that in the math/spatial teaching task, there is a significant positive correlation between sibling relationship quality and the learner’s verbal expressions (*r* = 0.53, *p*<0.01), as well as a significant positive correlation between sibling relationship quality and the learner’s physical expressions (*r* = 0.49, *p* < 0.01). In the language teaching task, there is a positive correlation between sibling relationship quality and the learner’s verbal expressions (*r* = 0.36, *p* < 0.05).

**Table 7:** The relationship between sibling relationship quality and learner’s behavior.

Task type	Predictor	Verbal expression	Physical expression	Refusal	Positive feedback	Negative feedback	Off-task
Math	SRQ	0.53**	0.49**	0.12	0.33	−0.25	0.09
Language	SRQ	0.36*	0.18	−0.12	0.17	−0.22	0.09

#### 3.5.2 The predictive role of sibling relationship quality on learner’s behavior

In both mathematics/spatial teaching tasks and language teaching tasks, the learner behaviors that are correlated with sibling relationship quality are entered into regression equations. In the mathematics/spatial teaching tasks, sibling relationship quality is used as the predictor variable, and the learner’s verbal expression and physical expression are the dependent variables. In the language teaching tasks, sibling relationship quality is used as the predictor variable, and the learner’s verbal expression is the dependent variable. Multiple linear regression analysis is conducted, and the results are presented in Table 8. The following conclusions can be drawn:

**Table 8:** The predictive role of sibling relationship quality on learner’s behavior.

Task type	Predictor variable	Dependent variable	*β*	*T*	*△R^2^*	*F*
Math	SRQ	Verbal expression	0.53	3.53**	0.28	12.47**
	SRQ	Physical expression	0.49	3.18**	0.24	10.08**
Language	SRQ	Verbal expression	0.36	2.18*	0.13	4.73*

In the mathematics/spatial teaching tasks, the predictor variable of sibling relationship quality can explain 28% of the variance in the learner’s verbal expression, *F* = 12.47, *p* < 0.01. Sibling relationship quality has a significant positive predictive effect on the learner’s verbal expression (*β* = 0.53, *T* = 3.53, *p* < 0.01). Thus, higher sibling relationship quality is associated with a greater frequency of verbal expression by the learner. The predictor variable of sibling relationship quality can explain 24% of the variance in the learner’s physical expression, *F* = 10.08, *p* < 0.01. Sibling relationship quality has a significant positive predictive effect on the learner’s physical expression (*β* = 0.49, *T* = 3.18, *p* < 0.01). Therefore, higher sibling relationship quality is associated with a greater frequency of physical expression by the learner.

In the language teaching tasks, the predictor variable of sibling relationship quality can explain 13% of the variance in the learner’s language expression, *F* = 4.73, *p* < 0.05. Sibling relationship quality has a significant positive predictive effect on the learner’s language expression (*β* = 0.36, *T* = 2.18, *p* < 0.05). Thus, higher sibling relationship quality is associated with a greater frequency of language expression by the learner. Taken together, these results indicate that higher sibling relationship quality was positively associated with learners’ verbal and physical engagement during sibling teaching, with similar patterns observed across both mathematics/spatial and language teaching tasks.

## 4 Discussion

### 4.1 The impact of theory of mind on instructors’ and learners’ behavior

Previous studies have consistently shown that sibling teaching is closely related to children’s social–cognitive development, instructional understanding, and interactional contexts (McAlister and Peterson, 2006; Howe et al., 2006; Howe and Funamoto, 2012; Recchia et al., 2009; Strauss and Ziv, 2012; Abuhatoum et al., 2016; Segal et al., 2018). Although several findings of the present study are consistent with prior research on sibling teaching, the current results extend existing accounts in ways that go beyond simple cultural replication. By distinguishing between different types of teaching behaviors, the findings suggest that children’s theory of mind is more strongly related to learner-centered and cognitively demanding instructional strategies, rather than to teaching behaviors in general (Ziv and Frye, 2004; Strauss and Ziv, 2012). In addition, the comparison between mathematical/spatial and language teaching activities highlights task type as a potential boundary condition, indicating that social–cognitive and relational mechanisms of sibling teaching may be differentially expressed across instructional contexts (Howe et al., 2006; Howe and Funamoto, 2012).

The differential patterns observed across mathematical/spatial and language teaching tasks further suggest that task structure and normative expectations may shape the extent to which social–cognitive and relational factors are activated during sibling teaching. In particular, instructional activities that place greater demands on problem solving and step-by-step guidance may be more sensitive to individual differences in social–cognitive abilities and relational context than tasks that rely more heavily on routine verbal interaction (Howe et al., 2006, 2016). These findings imply that sibling teaching behaviors are not uniform across contexts, but may vary as a function of both cognitive demands and situational expectations.

During teaching activities, children form an understanding of instructional goals and their own roles within the interaction. When children become aware that they possess greater knowledge than another child, they may recognize their potential role as a “little teacher,” leading to the emergence of intentional teaching behavior (Ziv and Frye, 2004). Previous research has shown that children as young as 3 years old can engage in teaching activities, and that with increasing age, children adopt more complex and abstract instructional strategies (Ziv et al., 2016; Segal et al., 2018). The development of second-order theory of mind has been proposed as an important cognitive foundation for this progression (McAlister and Peterson, 2006; Davis-Unger and Carlson, 2008), as it supports children’s ability to consider learners’ knowledge states and instructional needs. These developmental accounts help explain why, in the present study, theory of mind was particularly associated with the use of cognitively demanding and learner-centered teaching strategies, rather than with teaching behaviors more broadly.

#### 4.1.1 The impact of theory of mind on instructor’ behavior

In research focusing on sibling teaching activities, instructors’ theory of mind has been consistently associated with more learner-centered and adaptive instructional strategies (Howe and Funamoto, 2012; Strauss and Ziv, 2012; Ziv et al., 2016). Ziv et al. (2016) argue that a higher level of theory of mind enables instructors to better understand instructional dynamics, particularly the ability to adapt teaching strategies to learners’ actual needs and to select strategies that are most appropriate for the learner.

Previous studies have shown that instructors with higher levels of theory of mind tend to employ more cognitive strategies and provide more positive feedback during teaching interactions (Howe, 2004; Recchia et al., 2009; Howe et al., 2016, 2019). Ziv et al. (2008) further emphasize the role of second-order theory of mind, suggesting that the ability to consider learners’ beliefs and understanding allows instructors to adjust their teaching strategies in a timely and flexible manner, such as offering more detailed explanations or scaffolding when necessary. Instructors who possess these advanced social–cognitive abilities are therefore more likely to adopt learner-centered instructional approaches (Howe and Funamoto, 2012).

Consistent with this body of research, the present study found that, in both mathematical/spatial and language teaching tasks, instructors who scored higher on theory of mind measures used more cognitive strategies and provided more positive feedback during sibling teaching. Rather than reflecting a general increase in all teaching behaviors, this pattern suggests that theory of mind is particularly relevant for instructional actions that require sensitivity to learners’ understanding and the coordination of teaching goals with learners’ cognitive states.

Theory of mind refers to the capacity to understand and interpret others’ mental states, including their knowledge, beliefs, emotions, and intentions (Ziv et al., 2016). As children’s theory of mind develops, their ability to adopt others’ perspectives becomes more refined, which may facilitate the use of instructional strategies that are responsive to learners’ progress. In sibling teaching contexts, this is reflected in instructors’ use of encouragement, praise, and cognitively oriented guidance when learners successfully acquire new skills or concepts.

Within the Chinese family context, where older siblings are often expected to assume a guiding or instructional role, such social–cognitive abilities may be particularly important for adapting teaching behaviors to the needs of younger siblings. At the same time, an alternative explanation is that children with more advanced verbal abilities may also be more inclined to engage in cognitively demanding instructional strategies. Future research could further disentangle the unique contributions of social–cognitive understanding and language ability in shaping instructors’ teaching behaviors.

Notably, theory of mind was not uniformly associated with all forms of teaching behavior (Strauss and Ziv, 2012). This pattern challenges accounts that conceptualize teaching competence as a unitary construct and instead supports a more differentiated view, in which social–cognitive skills are especially relevant for learner-centered and cognitively demanding instructional strategies, rather than for teaching behaviors more broadly.

#### 4.1.2 The impact of theory of mind on and learners’ behavior

The acquisition of theory of mind equips children with the psychological capacity to understand others’ emotions, intentions, and goals, allowing them to interpret and anticipate others’ behavior during social interactions (Baron-Cohen et al., 1985; Gopnik and Astington, 1988; Wellman and Liu, 2004). In the present study, learners with higher levels of theory of mind exhibited fewer off-task behaviors in mathematical/spatial teaching activities and showed lower levels of refusal and disengagement in language teaching tasks. These findings suggest that learners’ theory of mind may play an important role in supporting behavioral regulation during instructional interactions.

One possible explanation is that children with more advanced theory of mind are better able to recognize the instructor’s intention to provide help and guidance, which may reduce misunderstandings and resistance during teaching (Ziv et al., 2008; Abuhatoum et al., 2016). As a result, such learners are less likely to disengage, ignore instructional cues, or become distracted, and are more likely to remain attentive and responsive to instructional guidance. In this sense, theory of mind may facilitate learners’ participation in teaching interactions by supporting their understanding of the interpersonal goals underlying instructional behavior (Davis-Unger and Carlson, 2008).

Importantly, although theory of mind was associated with reduced off-task and disengagement behaviors, it did not predict learners’ verbal expression during teaching activities. This pattern suggests that behavioral regulation and expressive participation may rely on partially distinct mechanisms (Liu and Li, 2010; Liu et al., 2008). While theory of mind may primarily support learners’ ability to regulate attention and interpret instructional intentions, verbal expression during teaching interactions may be more strongly influenced by factors such as language proficiency, task familiarity, or individual communicative style. These findings highlight the need to conceptualize learners’ participation in sibling teaching as a multidimensional process, in which social–cognitive understanding contributes selectively to certain aspects of engagement rather than uniformly shaping all observable behaviors (Howe and Recchia, 2009; Howe et al., 2019).

### 4.2 The impact of sibling relationship quality on instructors’ and learners’ behavior

#### 4.2.1 The impact of sibling relationship quality on instructors’ behavior

When siblings share a warmer and more positive relationship, they are more likely to engage in frequent interactions, which in turn provides greater opportunities for mutual observation and learning (Brody, 1998). Research has shown that the quality of sibling interactions established during early childhood lays an important foundation for later patterns of cooperation and engagement (Howe and Recchia, 2009). Consistent with this perspective, previous studies have reported that instructors embedded in higher-quality sibling relationships tend to adopt more cognitively oriented and learner-centered teaching strategies (Howe, 2004; Recchia et al., 2009; Howe et al., 2016, 2019).

The findings of the present study align with prior research by showing that, across both mathematical/spatial and language teaching tasks, higher sibling relationship quality was associated with greater use of cognitive strategies by instructors. One possible explanation is that positive sibling relationships facilitate a cooperative interactional climate, in which instructors are more willing to invest effort in explaining, guiding, and scaffolding learners’ understanding (Howe and Recchia, 2005; Howe and Funamoto, 2012). In this sense, sibling relationship quality may function as a contextual resource that supports instructors’ engagement in cognitively demanding teaching behaviors.

In addition, sibling relationship quality was negatively associated with instructors’ use of negative feedback in mathematical/spatial teaching tasks. Mathematical and spatial tasks often require step-by-step guidance, sustained attention, and error correction, which may increase the likelihood of frustration during instruction (Howe et al., 2006, 2016). In the context of a positive sibling relationship, instructors may be better able to regulate negative emotions and respond to learners’ difficulties with patience rather than criticism (Brody, 1998). As a result, higher-quality sibling relationships may buffer against the use of negative feedback in instructional situations that place greater cognitive and emotional demands on instructors.

Notably, sibling relationship quality did not emerge as a significant predictor of all instructional behaviors across tasks. One possible explanation is that, within Chinese families, parental norms emphasizing compliance, responsibility, and older siblings’ instructional roles may partially override relational dynamics in certain contexts (Zhao and Yu, 2017). Under such normative expectations, instructors may continue to engage in teaching behaviors even when sibling relationships are less warm, thereby reducing the observable influence of relationship quality in some instructional situations.

#### 4.2.2 The impact of sibling relationship quality on learners’ behavior

Sibling relationship quality plays an important role in shaping learners’ engagement during sibling teaching activities. A higher-quality sibling relationship provides a supportive interpersonal context in which learners are more willing to observe, imitate, and actively participate in instructional interactions (Brody, 1998). Previous research has shown that learners who perceive sibling relationships more positively are more likely to adopt imitation and cooperative learning strategies during teaching activities (Howe and Recchia, 2009). Although first-born children typically assume the role of instructors due to greater knowledge and experience, the present findings underscore the importance of second-born children’s perceptions of sibling relationship quality in fostering their active involvement as learners (Howe et al., 2015; 2019).

One possible explanation is that higher-quality sibling relationships promote relational alignment and trust between siblings, making learners more receptive to instructional guidance (Brody, 1998; Howe and Recchia, 2005). When learners feel supported and emotionally secure within the sibling relationship, they may be more willing to follow instructors’ directions and sustain engagement during teaching tasks. In mathematical/spatial teaching activities, such engagement is reflected in increased use of both verbal and physical expressions, which are often required to demonstrate understanding, manipulate materials, or follow step-by-step guidance (Howe et al., 2006, 2016). In contrast, language teaching tasks rely more heavily on verbal responses, and learners’ engagement in these tasks may therefore be expressed primarily through language-based participation (Segal et al., 2018).

These findings suggest that sibling relationship quality contributes to learners’ active participation by shaping the interpersonal conditions under which learning takes place. Rather than uniformly increasing all forms of learner behavior, relationship quality appears to influence the specific modes of engagement that are most relevant to the instructional demands of different task types. This task-sensitive pattern further supports the view that sibling teaching is a context-dependent process, jointly shaped by relational dynamics and instructional characteristics.

### 4.3 Limitations and future directions

Despite the strengths of the observational design, several limitations of the present study should be acknowledged, which also point to directions for future research. First, the relatively small sample size of 34 sibling dyads may limit the generalizability of the findings. Although this sample size is comparable to that of prior observational studies on sibling interactions, the results should be interpreted with caution and may not readily extend to broader populations or different cultural contexts. Future research with larger and more diverse samples would be valuable for replicating and extending the present findings.

Second, the study focused on structured teaching tasks within a specific observational context, which may not fully capture the range of sibling teaching behaviors that occur in everyday family interactions. Teaching dynamics in naturalistic settings may differ in important ways from those observed in task-based situations. Future studies could adopt longitudinal or naturalistic designs to examine how theory of mind and sibling relationship quality influence sibling teaching across a wider range of daily contexts.

Finally, although the present study examined both instructors’ and learners’ behaviors, other potentially relevant factors—such as parental involvement, family educational practices, or individual language abilities—were not directly assessed. Future research could incorporate these variables to develop a more comprehensive understanding of the mechanisms underlying sibling teaching processes.

## 5 Conclusion

This study examined the roles of theory of mind and sibling relationship quality in shaping instructors’ and learners’ behaviors during sibling teaching within Chinese families with two children. The findings indicate that theory of mind is particularly relevant for cognitively demanding and learner-centered instructional strategies, as well as for learners’ behavioral regulation during teaching interactions. In addition, sibling relationship quality emerged as an important contextual factor that supports both instructors’ and learners’ engagement, with its influence varying across different instructional tasks. Together, these results highlight sibling teaching as a context-dependent process jointly shaped by social–cognitive development and relational dynamics. The present study contributes to a more differentiated understanding of sibling teaching and underscores the importance of considering both cognitive and relational factors when supporting positive learning interactions between siblings.

## Data Availability

The raw data supporting the conclusions of this article will be made available by the authors, without undue reservation.

## Supplementary material

Appendix A Descriptions of the mathematical/spatial teaching tasks (simple and complex tasks).

Appendix B Descriptions of the language teaching tasks (simple and complex tasks).

Appendix C Detailed instruments used to assess Theory of Mind in firstborn and second-born children.

Appendix D Parent-reported questionnaire on sibling relationship quality.
