# Supplementary material for: The influence of children’s theory of mind and sibling relationship quality on sibling teaching in the Chinese family with two children
Source: Front Psychol. 2026 Feb 11;17:1751491. doi: 10.3389/fpsyg.2026.1751491 (PMC12932414; doi:10.3389/fpsyg.2026.1751491)
Supplement: Appendix A — Descriptions of the mathematical/spatial teaching tasks (simple and complex tasks). [file Table_1.DOCX]

**Appendix A**

**Math/Spatial Teaching Task**

(1) Simple Teaching Task


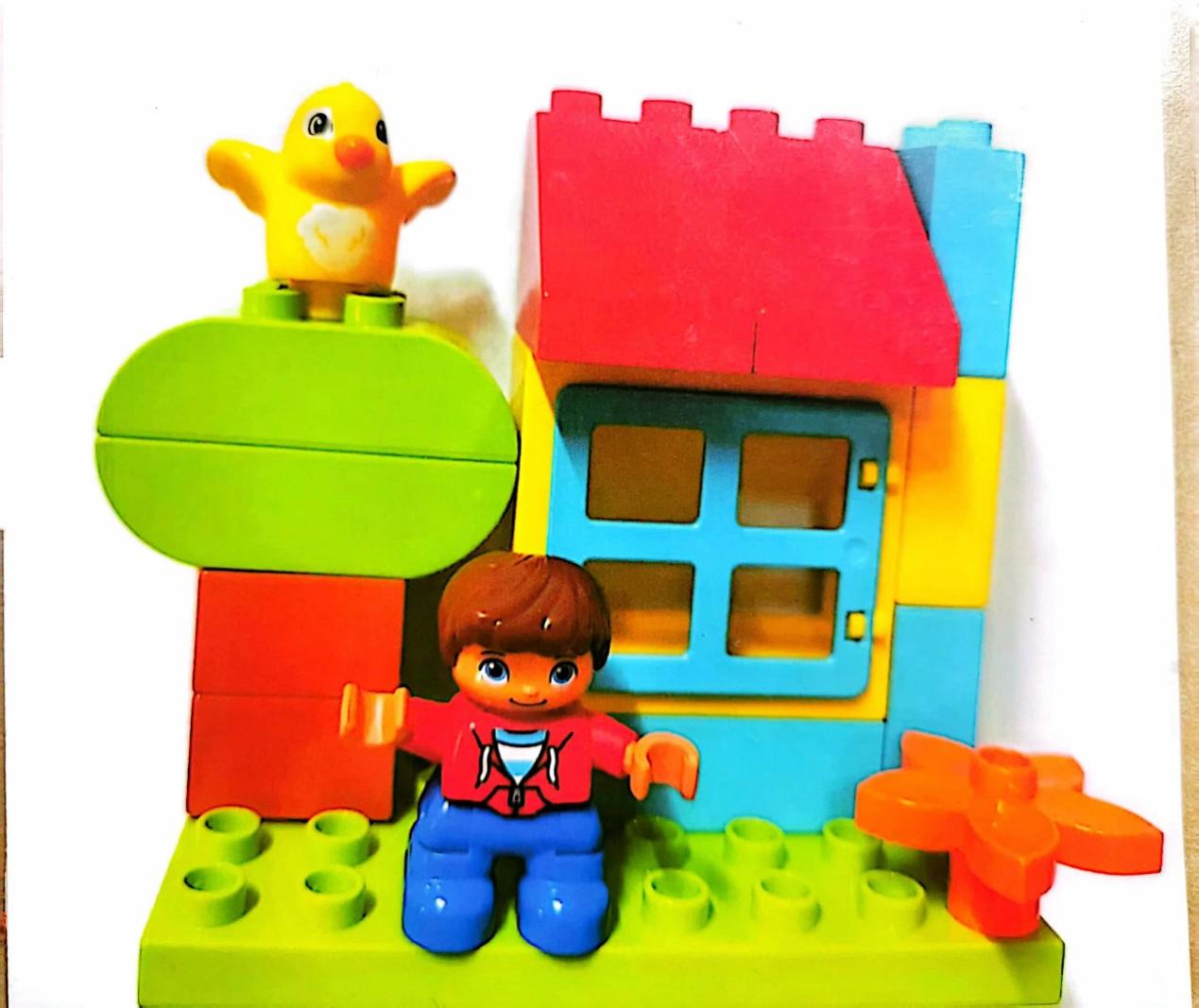


(2) Complex Teaching Task


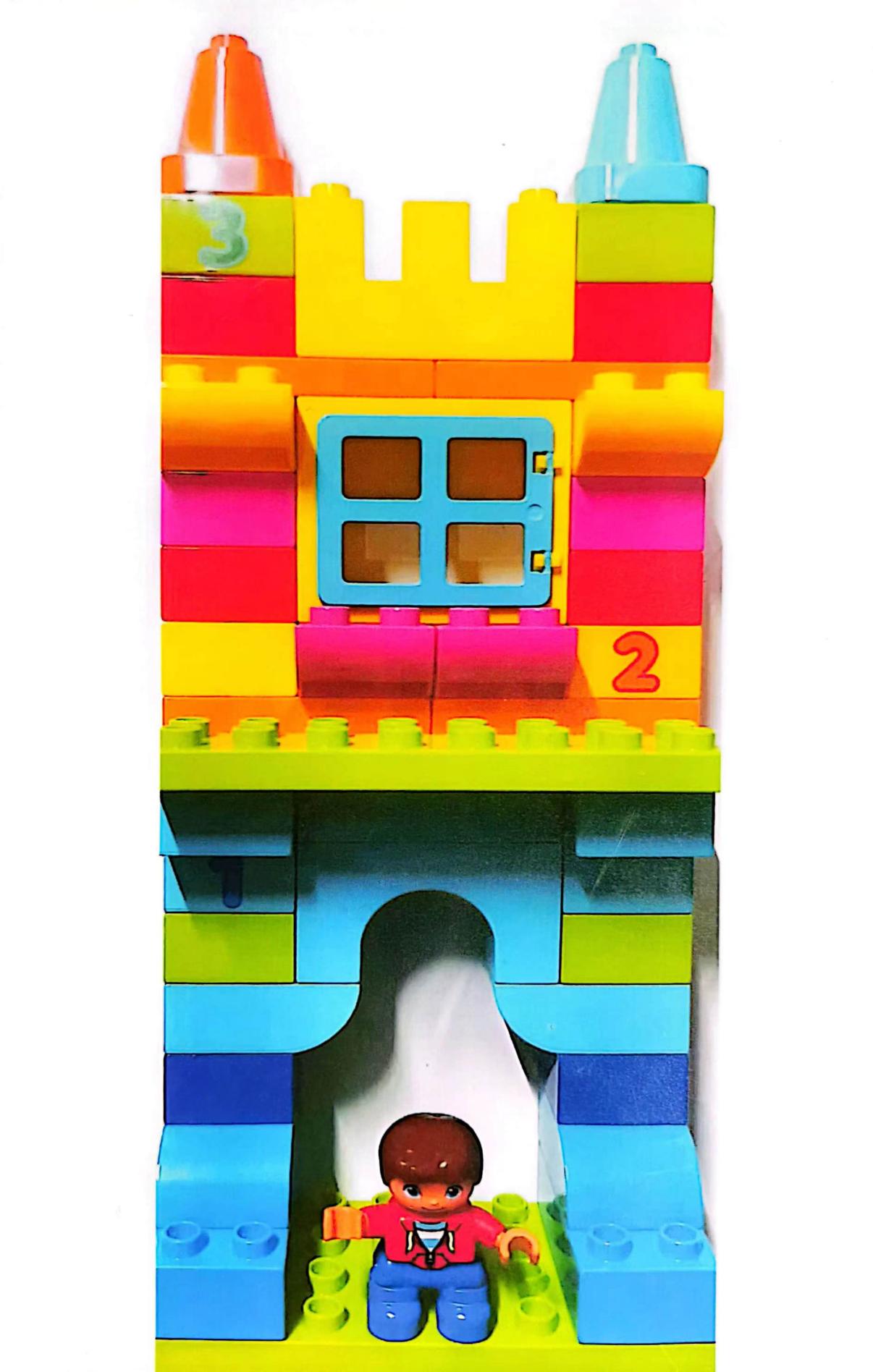


**Appendix B**

**Language Teaching Task**

(1) Simple Teaching Task


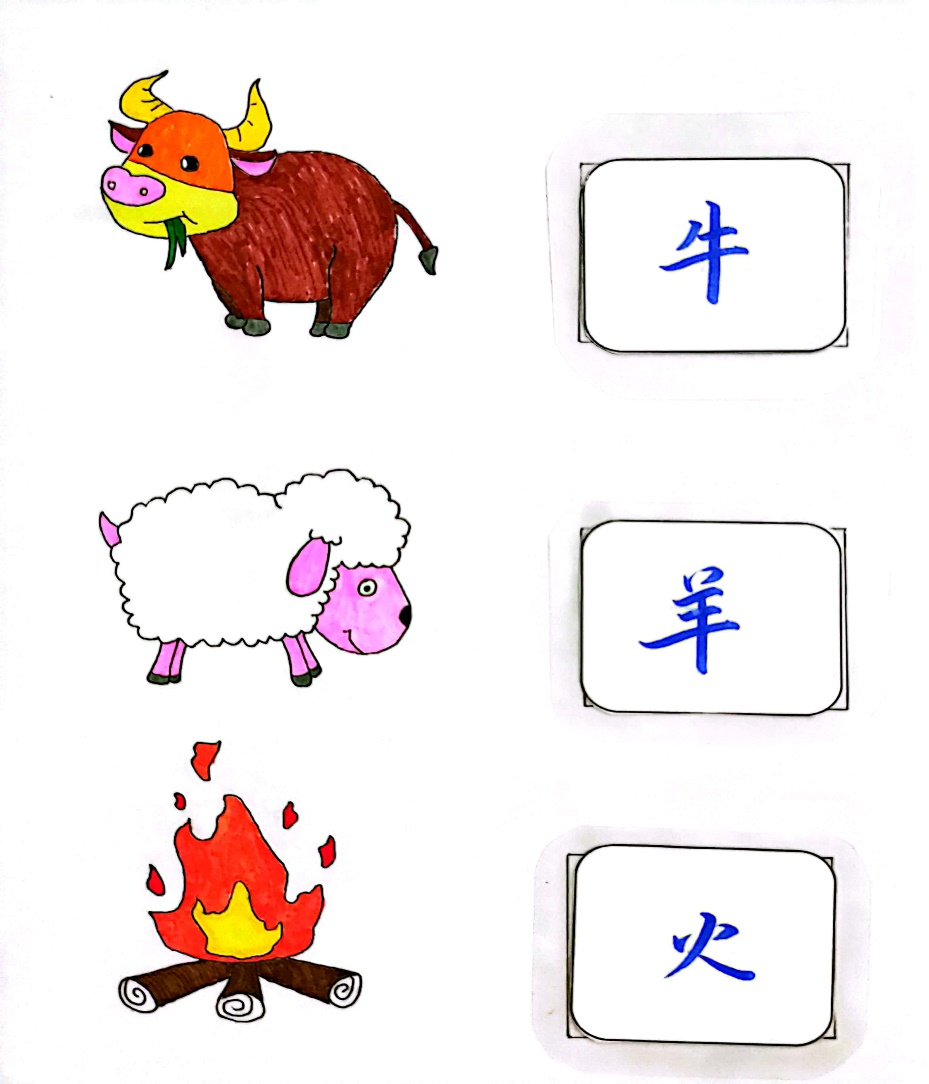


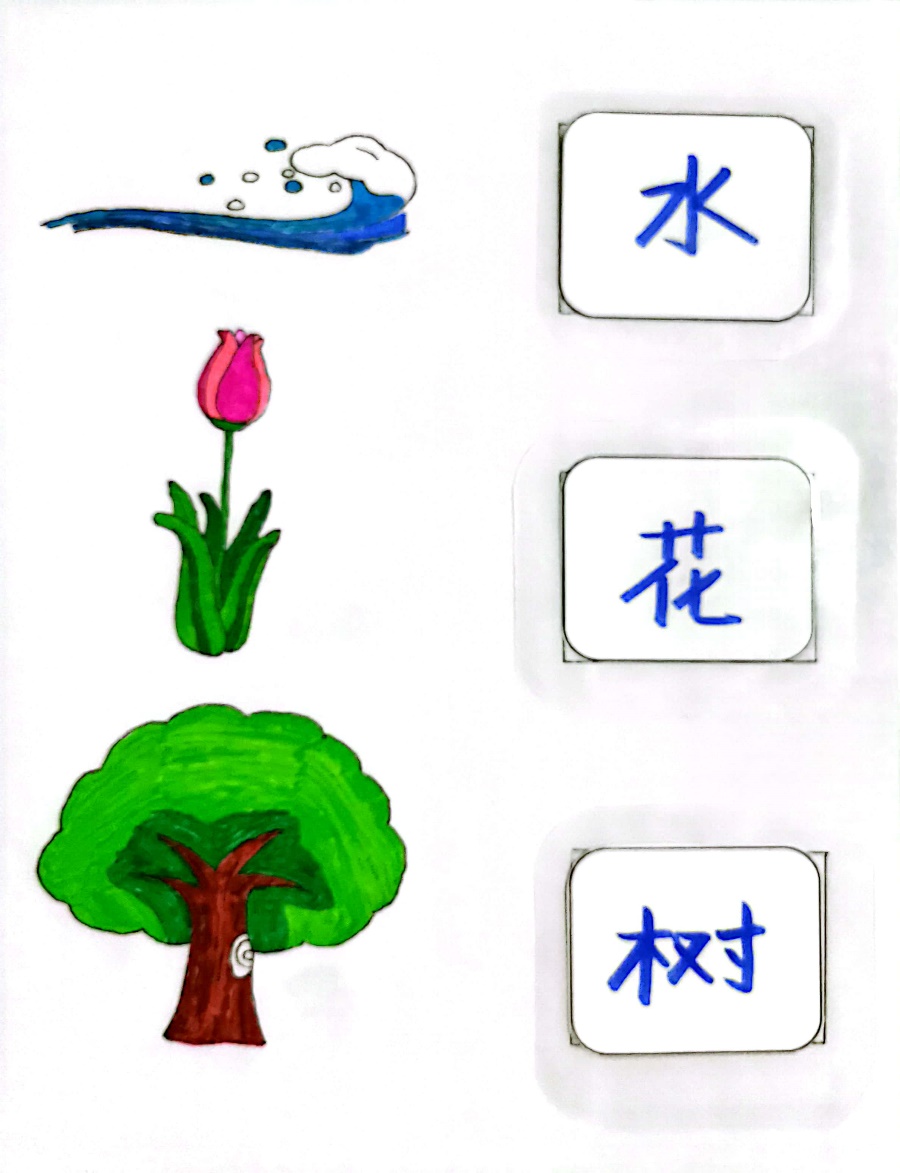


(2) Complex Teaching Task


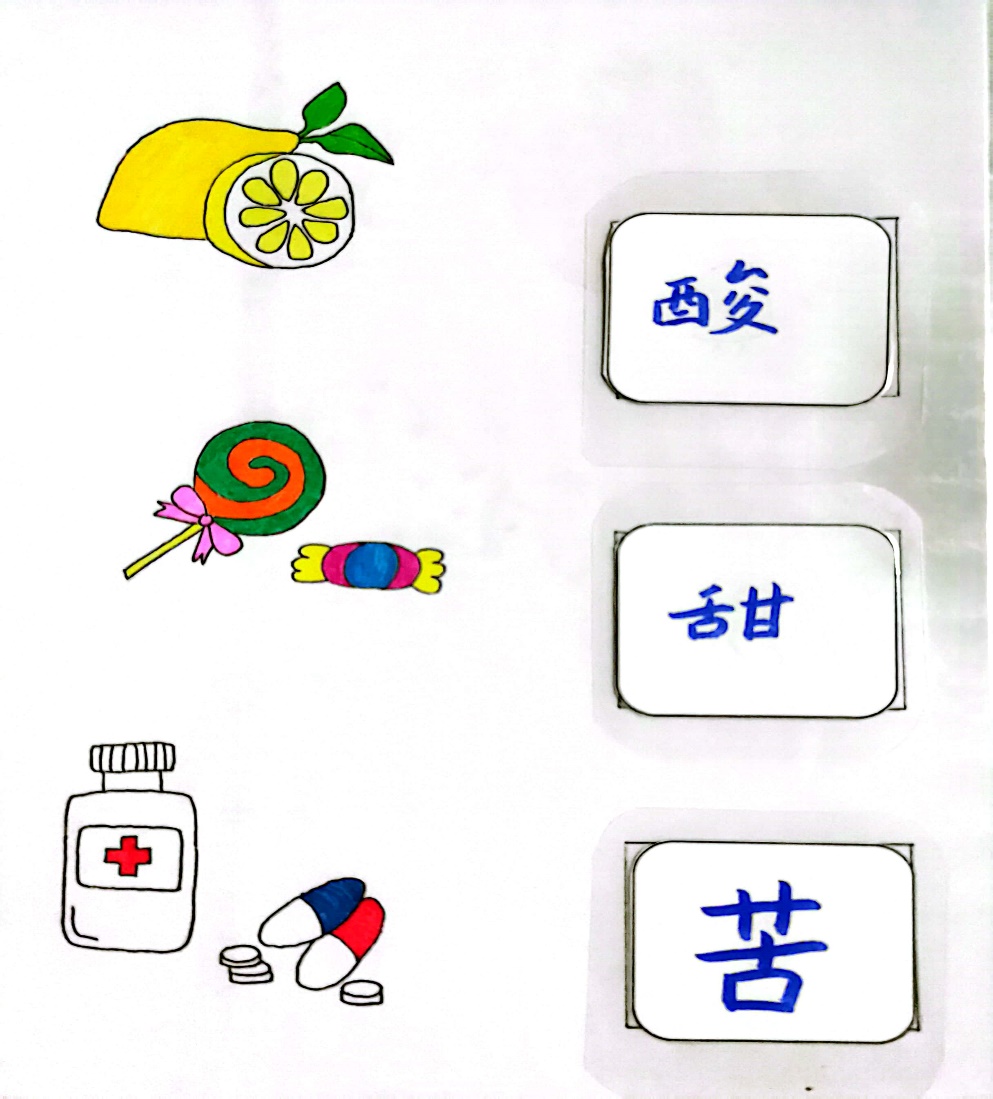


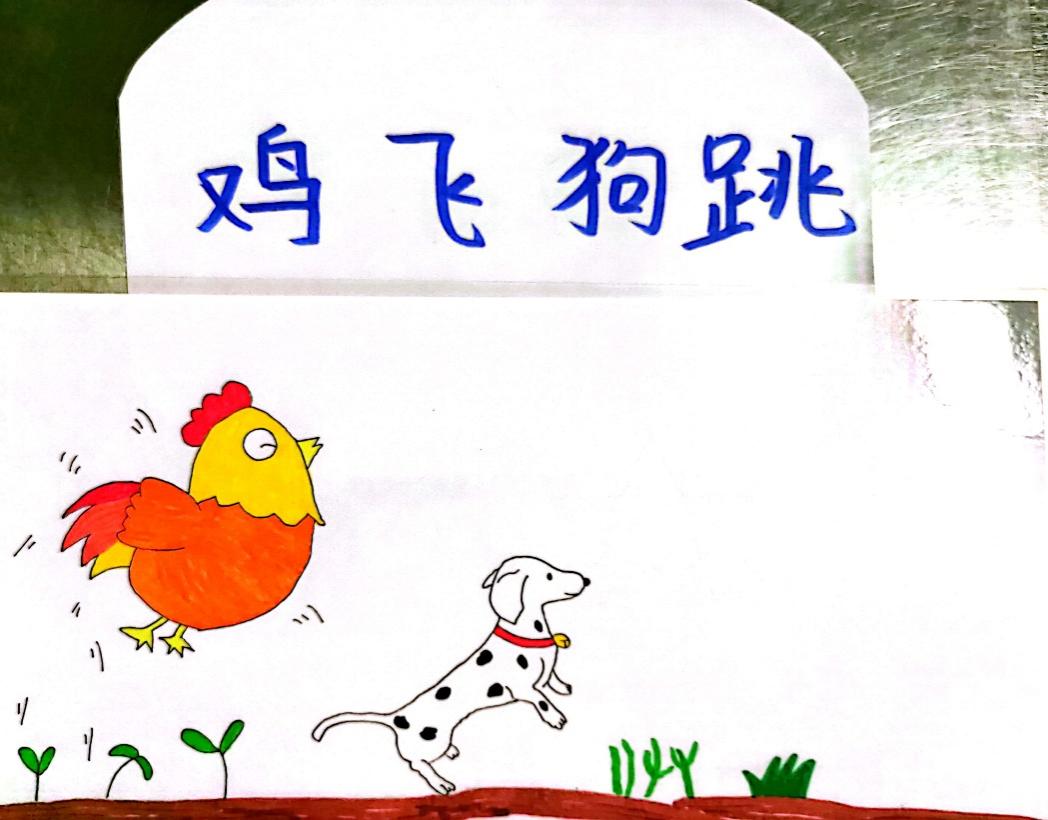


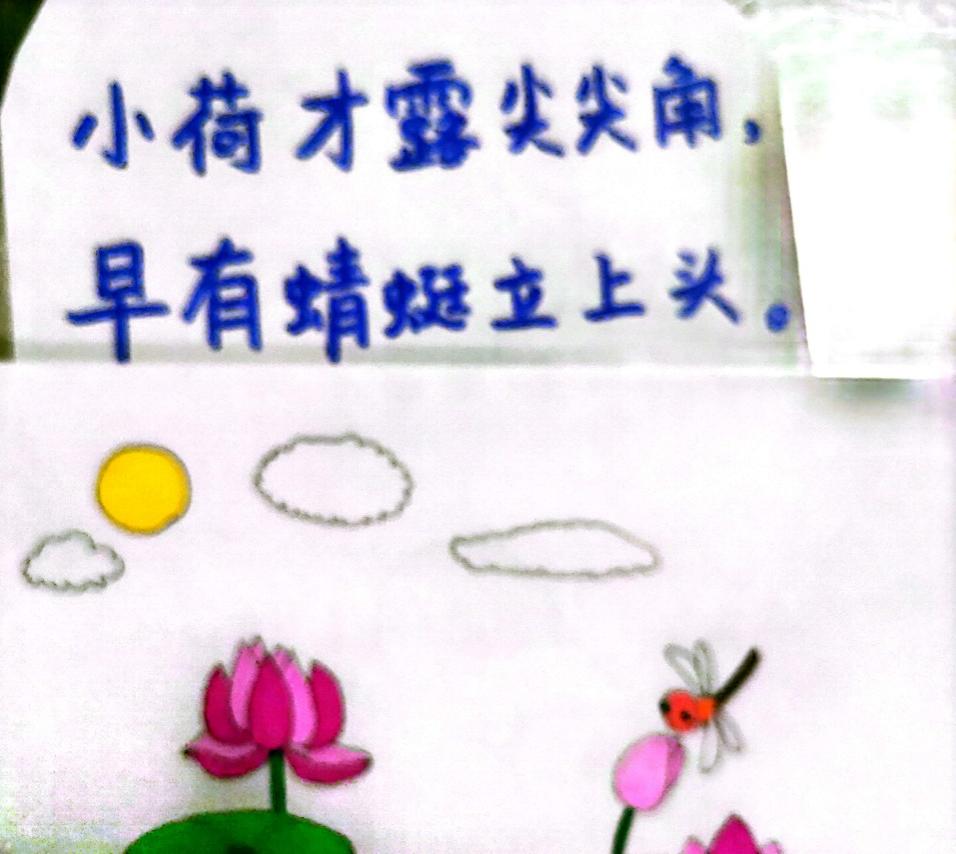


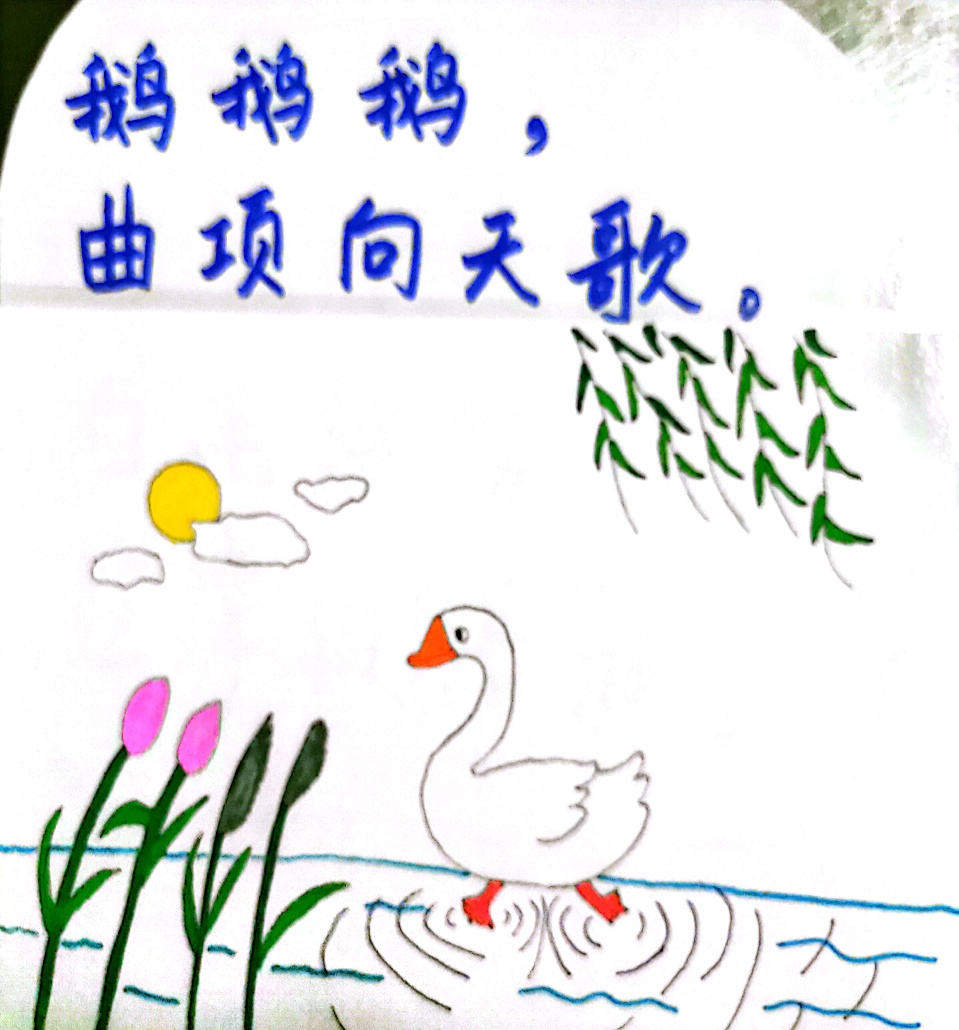


**Appendix C**

First, the test tool for assessing the theory of mind in the firstborn child consists of two parts, with a total of 5 tasks:

**(1) Belief-Desire Reasoning Task:**

**①Story 1 (Second-Level True Belief - Proximity Desire)**: Xiao Ming and Xiao Fang both like lollipops. They put the lollipop in a red box together. Then, Xiao Ming’s mother calls Xiao Ming outside, and while Xiao Ming is away, Xiao Fang takes the lollipop out of the red box and puts it in a green box. Xiao Ming returns. The child is asked:

(a) Two Control Questions: Where was the lollipop originally placed? Where was the lollipop moved to later?

(b) One Desire Question: Does Xiao Ming want the lollipop?

(c) Second-Level Belief-Desire Reasoning Question: Where does Xiao Fang think Xiao Ming will look for the lollipop?

(d) Confirmation Question: Why does Xiao Fang think that?

**②Story 2 (Second-Level True Belief - Avoidance Desire)**: There is a red house and a green house, as well as a playground. There is a dog in the red house, and both Xiao Ming and Xiao Fang are afraid of the dog, so they play in the green house. After a while, they go to play on the playground. At this time, Xiao Ming’s mother calls him away. While Xiao Ming is gone, Xiao Fang sees the dog run from the red house to the green house. After a while, Xiao Ming returns to the playground and tells Xiao Fang that he wants to play inside the house. The child is asked:

(a) Two Control Questions: Where was the dog originally located? Where did the dog move to later?

(b) One Desire Question: Does Xiao Ming want to play in the house with the dog or in the house without the dog?

(c) Second-Level Belief-Desire Reasoning Question: Which house does Xiao Fang think Xiao Ming will go to play in?

(d) Confirmation Question: Why does Xiao Fang think that?

**③Story 3 (Second-Level False Belief - Proximity Desire)**: Xiao Ming and Xiao Fang both like playing with lollipops. They put the lollipop in a red box together. Then Xiao Ming’s mother calls him out, and while Xiao Ming is away, Xiao Fang takes the lollipop out of the red box and puts it in the green box. When Xiao Ming returns, he sees Xiao Fang putting the lollipop into the green box, but Xiao Fang does not see Xiao Ming. The child is asked:

(a) Two Control Questions: Where was the lollipop originally placed? Where was the lollipop moved to later?

(b) One Desire Question: Does Xiao Ming want the lollipop?

(c) Second-Level Belief-Desire Reasoning Question: Where does Xiao Fang think Xiao Ming will look for the lollipop?

(d) Confirmation Question: Why does Xiao Fang think that?

**④Story 4 (Second-Level False Belief - Avoidance Desire)**: There is a red house and a green house, as well as a playground. There is a dog in the red house, and both Xiao Ming and Xiao Fang are afraid of the dog, so they play in the green house. After a while, they go to play on the playground. At this time, Xiao Ming’s mother calls him away. While Xiao Ming is gone, Xiao Fang sees the dog run from the red house to the green house. However, when Xiao Ming returns, he sees the dog running from the red house to the green house. Xiao Fang does not see Xiao Ming. Xiao Ming returns to the playground and tells Xiao Fang that he wants to play inside the house. The child is asked:

(a) Two Control Questions: Where was the dog originally located? Where did the dog move to later?

(b) One Desire Question: Does Xiao Ming want to play in the house with the dog or in the house without the dog?

(c) Second-Level Belief-Desire Reasoning Question: Which house does Xiao Fang think Xiao Ming will go to play in?

(d) Confirmation Question: Why does Xiao Fang think that?

**(2) Misstatement Understanding Task**:

**⑤**Story 5: Chen Chen gave his good friend Guo Guo a glass as a birthday gift. At the time, Guo Guo received many birthday presents and couldn’t remember who gave which gift. A few days later, Chen Chen visited Guo Guo again and accidentally broke the glass he had given. Guo Guo said, "It’s okay, I didn’t like this glass anyway; it was just a birthday gift someone gave me on my birthday." The child is asked:

(a) Was there a misstatement? (This question assesses awareness of the misstatement situation).

(b) Who made the misstatement? (This question examines understanding of the misstatement situation).

(c) Why should he/she not have said that? (This question requires understanding of the listener's mental state).

(d) Why did he/she say that? (This question requires understanding of the speaker's mental state).

Second, the test tool for assessing the theory of mind in second-born children consists of 3 tasks, with a final score ranging from 0 to 6 points.

(1) **Unexpected Location Task**:

Story and Task: Lily has a basket and a ball, and An An has a box. Lily puts the ball into the basket in front of An An and then leaves. An An takes the ball out of the basket and puts it into the box. The child is asked:

(a) Detection Questions: Where was the ball before Lily left? Where is the ball now?

(b) False Belief Question: Where does Lily think the ball is?

(c) Behavior Prediction Question: Where will Lily look for the ball when she comes back?

(2) **Unexpected Content Task**:

The researcher shows the child a toothpaste box and asks what is inside it. The researcher then opens the box and takes out a pencil, puts the pencil back in the box, and closes the lid. The child is asked:

(a) Detection Question: What is inside the box?

(b) Representation Change Question: Before I opened the box, what did you think was inside?

(c) False Belief Question: If a friend came in and looked at the box without seeing the contents, what do you think he/she would think is inside?

(3) **Appearance-Reality Distinction Task**:

The researcher presents a realistic plastic apple to the child and asks them to identify it. After the child identifies it as an apple, the researcher allows the child to look at and touch the plastic apple. The child is asked:

(a) Detection Question: Can this apple be eaten?

(b) Representation Change Question: Before you touched it, what did you think this was?

(c) False Belief Question: If a friend came in, looked at this apple without touching it, what do you think he/she would think it is?

**Appendix D**

Please answer the following questions according to the actual situation of your children, and mark (√) on the appropriate number.

(Choose only one option for each question, please do not select multiple options or omit any)

1.Basic Information about Your Children (Elder Sibling and Younger Sibling):

(1) Gender of Elder Sibling: Male Female

Age of Elder Sibling:

(2) Gender of Younger Sibling: Male Female

Age of Younger Sibling:

2. Questionnaire Information

**
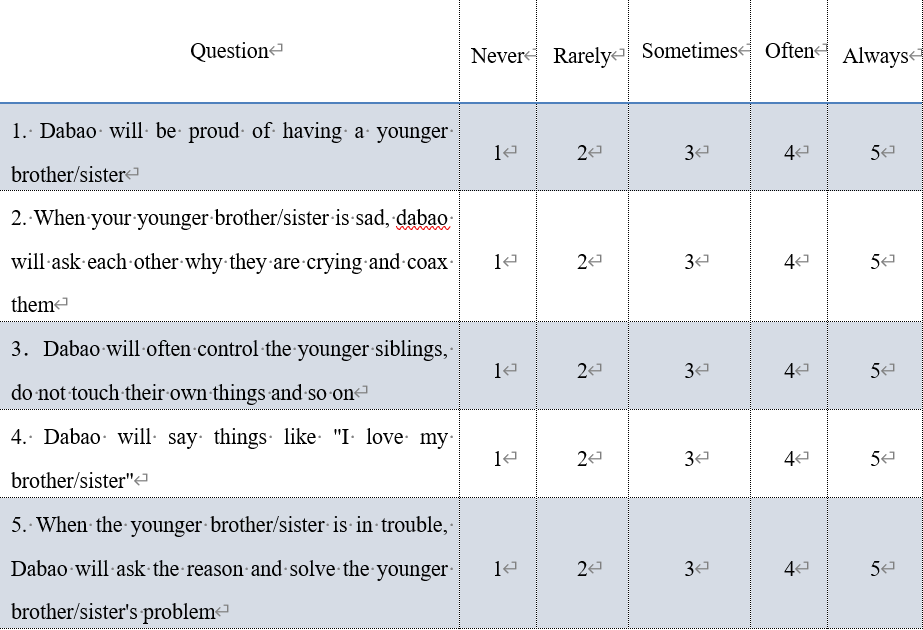
**

**
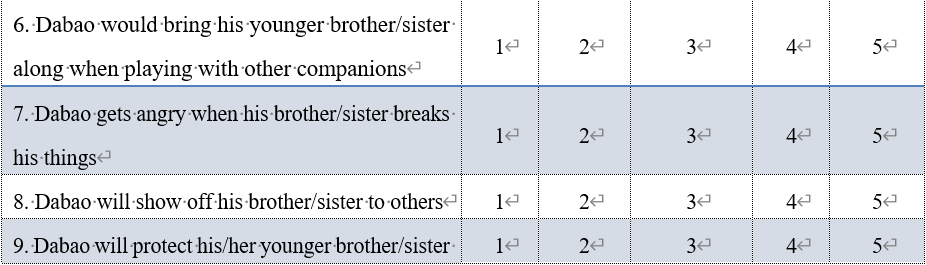
**

**
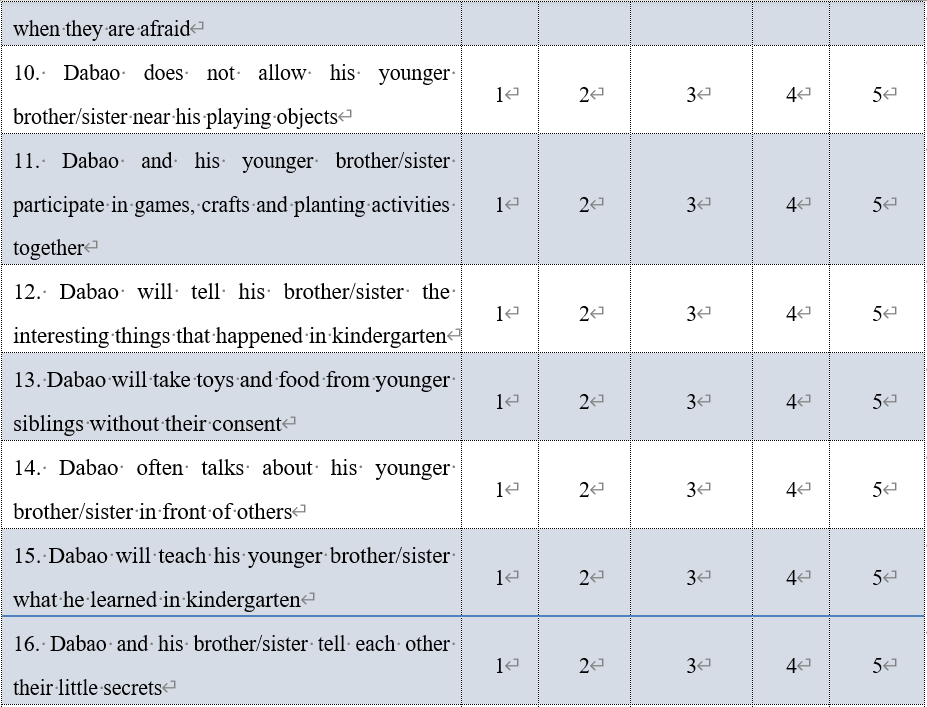
**

**
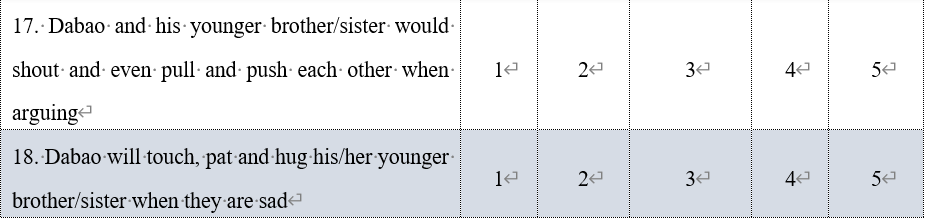
**
